# Ghrelin/GHSR System in Depressive Disorder: Pathologic Roles and Therapeutic Implications

**DOI:** 10.3390/cimb46070434

**Published:** 2024-07-10

**Authors:** Xingli Pan, Yuxin Gao, Kaifu Guan, Jing Chen, Bingyuan Ji

**Affiliations:** 1 School of Biological Sciences, Jining Medical University, Jining 272067, China; xingli7783@163.com; 2 School of Clinical Medicine, Jining Medical University, Jining 272067, China; gyxxx_11@163.com (Y.G.); kaifu_guan@163.com (K.G.); 3 Neurobiology Institute, Jining Medical University, Jining 272067, China; 4 Division of Biomedical Sciences, Warwick Medical School, University of Warwick, Coventry CV4 7AL, UK; 5 Institute of Precision Medicine, Jining Medical University, Jining 272067, China

**Keywords:** GPCR, ghrelin, GHSR, cell signaling, depression

## Abstract

Depression is the most common chronic mental illness and is characterized by low mood, insomnia, and affective disorders. However, its pathologic mechanisms remain unclear. Numerous studies have suggested that the ghrelin/GHSR system may be involved in the pathophysiologic process of depression. Ghrelin plays a dual role in experimental animals, increasing depressed behavior and decreasing anxiety. By combining several neuropeptides and traditional neurotransmitter systems to construct neural networks, this hormone modifies signals connected to depression. The present review focuses on the role of ghrelin in neuritogenesis, astrocyte protection, inflammatory factor production, and endocrine disruption in depression. Furthermore, ghrelin/GHSR can activate multiple signaling pathways, including cAMP/CREB/BDNF, PI3K/Akt, Jak2/STAT3, and p38-MAPK, to produce antidepressant effects, given which it is expected to become a potential therapeutic target for the treatment of depression.

## 1. Introduction

Growth-hormone-releasing peptide (ghrelin) is a multifunctional 28-amino-acid peptide hormone which is mainly released from the stomach but is also expressed in a number of other tissues, including the pancreas and kidneys [1]. Ghrelin’s serine 3 (Ser3) is n-octastylated, a modification that allows for the cycling of both the acylated and des-acylated forms of ghrelin [2]. Ghrelin can bind to central growth-hormone-releasing peptide receptor (GHSR), which is widely found in the central nervous system (CNS) and peripheral tissues, and has two isoforms, GHSR1a and GHSR1b [3]. Ghrelin mainly binds to GHSR1a and exerts a variety of physiological and behavioral modulatory effects, such as the regulation of glucose homeostasis, the regulation of metabolism and energy homeostasis, the modulation of blood pressure, and renal protection [4,5,6]. GHSR1b contains 289 amino acids and is a splice variant and a dominantly inactivated form of GHSR1a [7]. Due to the absence of the sixth and seventh transmembrane chains, it is unable to bind ghrelin and does not have signaling ability. There is much evidence that GHSR1b does not bind ghrelin, but it can heterodimerize with GHSR1a to interfere with GHSR1a function [8]. In an experiment to study the expression of the two GHSR forms cloned from black seabream in HEK293 cells, GHSR1b was found to inhibit the signaling activity of the GHSR1a-mediated increase in intracellular Ca^2+^ concentration [9]. GHSR1a has also been shown to form mixed heterodimers with dopamine receptor subtype 2 (D2R) [10], serotonin 2c receptor (5-HT2cR) [11], and orexin 1 receptor (OX1R) [12], and the dimers can fine-tune its activity.

Depression is a prevalent severe chronic mental illness which is characterized by episodes of depressed mood, appetite disorders, sleep disturbances, bipolar disorder, and loss of interest and pleasure. The above-mentioned disorders may be attributed to decreased size and function of the hippocampus and amygdala in the limbic brain regions [13]. With the rise in survival pressures in contemporary society and the dramatic increase in prevalence and incidence in different parts of the country, depression is emerging as a complex disorder present in all parts of the population, including the pregnant population, the adolescent population, and the elderly population [14,15]. Although the exact pathogenesis of the disorder remains obscure, a large body of evidence strongly suggests that stress and genetic predisposition are collectively involved in the progression of the disorder [16]. The pathophysiologic processes of depression include inflammation in the brain, reduced neurogenesis, monoamine neurotransmitter changes, and endocrine abnormalities [17,18,19]. However, the interrelationships among these processes remain unclear. Currently, several classes of drugs are available for the treatment of major depressive disorder (MDD) [20], and most of them exert their antidepressant effects by enhancing monoaminergic function, such as typical monoamine oxidase inhibitors (MAOIs) and selective 5-hydroxytryptamine reuptake inhibitors (SSRIs) [21]. In addition, scientists are now working on research beyond typical monoamine targets and pathways to develop antidepressants with novel mechanisms of action [22].

However, the efficacy and severe side effects of these antidepressants remain major issues to be addressed [23], and there are no therapeutic candidates shown to completely eliminate disease progression. In line with ghrelin’s effects on the CNS, there is increasing evidence for an important role of ghrelin in depression [24,25,26]. The majority of research has elucidated the anti-depressive mechanisms of ghrelin. Conversely, a few studies have reported a detrimental effect as ghrelin itself induces depressive tendencies and patterns in animal models. Here, we attempted to elucidate the aforementioned link between ghrelin and the onset as well as the treatment of depression and to elucidate the mechanisms and pathways that may be involved in the development of depression. We performed such an explanatory study by conducting a comprehensive review of the existing literature (reviews and research articles) through keyword searches.

## 2. Mechanisms of Ghrelin/GHSR System in Depressive Disorder

Numerous studies have shown that the ghrelin/GHSR system inhibits key pathways and mechanisms in the development of depression that have been found to correlate with certain factors, transmitters, and cells in the body in a variety of models of depression, which we will describe in more detail in the following paragraphs.

### 2.1. Links between Monoamine Neurotransmitter Receptors and Ghrelin

The monoamine hypothesis, long considered to be the most common hypothesis for depression pathogenesis, is centered on the fact that the concentration of monoamines (5-hydroxytryptamine, norepinephrine, and dopamine) in the synaptic gap is reduced in depressive states [27]. These monoamine neurotransmitters may influence ghrelin’s neuropathology. It was found that in neurons co-expressing D1R and GHSR, ghrelin can amplify dopamine signaling by activating GHSR to enhance its mediated downstream pathways [28]. Moreover, the mRNA expression of dopaminergic receptors was reduced in the amygdala and dorsal raphe nucleus of GHSR1a−/− mice [29], suggesting the potential regulation of dopamine neuron production by the ghrelin/GHSR system. In addition to this, another study showed that the central 5-hydroxytryptamine system is a target of ghrelin [29], and increased mRNA expression of some 5-hydroxytryptamine receptors in the amygdala and the dorsal nucleus of the intermediate suture was found in mice acutely centrally administered with ghrelin. Conversely, unlike the ghrelin/GHSR system described above, which regulates the expression of monoamine neurotransmitters, norepinephrine has been shown to stimulate ghrelin secretion in mouse cells [30], and the depletion of catecholamine-secreting neurons can also inhibit fasting-induced ghrelin secretion [31]. Moreover, GHSR1a differentially inhibits presynaptic voltage-gated calcium channels (CaV2.1 and CaV2.2) via the Gi/o- or Gq-dependent pathway, which reduce GABA release from hypothalamic neurons [32], contributing to neuronal activation through the disinhibition of postsynaptic neurons. In conclusion, there are many unknown connections between the monoamine system and the ghrelin/GHSR system, and more research is needed to determine whether monoamine receptors can be used as targets in the treatment of depression.

### 2.2. The Ghrelin/GHSR System Mediates the Inflammatory Response to Depression

In the CNS, IL-1β stimulates microglia and astrocytes to produce other cytokines, such as IL-6 and TNF-α, to promote inflammation in the brain [33]. Furthermore, pro-inflammatory cytokines such as IL-1β and TNF-α have been reported to play an important role in the onset of depression [34], and an increase in pro-inflammatory cytokines contributes to the development of depression [17]. Moreover, IL-6 is the most persistently elevated cytokine in the blood of MDD patients; so, it may serve as a predictive biomarker and a potential target for the treatment of depression in humans [35]. These experimental conclusions show that the role of pro-inflammatory cytokines of the CNS in the pathogenesis of depression cannot be ignored.

It is well-documented that serum ghrelin concentration is positively correlated with inflammation. For instance, serum ghrelin concentrations were higher in rats with severe pancreatitis, arthritis, and acute colitis. Nonetheless, rheumatoid arthritis patients and arthritis rats had lower levels of serum ghrelin concentrations [36]. This discrepancy may be due to unpredictable reasons, such as rat nutritional status or experimental conditions, making it difficult to analyze the effect of inflammation on serum ghrelin levels. However, it is undeniable that ghrelin levels are elevated in inflammation, as demonstrated in most experiments. On the whole, the causal relationship between inflammation and changes in serum ghrelin concentrations requires further study, although the effects of various conditions cannot be ruled out. Pursuant to the existing data, there are many reports indicating that elevated serum ghrelin concentrations are the result of inflammation. Interestingly, experiments have found that elevated ghrelin levels are involved in regulating inflammatory response, downregulating neutrophil transport and the number of pro-inflammatory cytokines, significantly reducing cerebral ischemic injury, and improving neurobehavioral function [37]. The same study also reported that exogenous ghrelin inhibits the endothelial cell production of IL-1, IL-6, and IL-8 by regulating the release of pro-inflammatory cytokines, which play an important role in the pathologic process of depression [38]. In addition, GHSR and ghrelin are expressed in human T lymphocytes and monocytes, and ghrelin specifically inhibits the expression of pro-inflammatory cytokines via GHSR [39]. In addition, two other investigations displayed decreased expression of pro-inflammatory cytokines such as TNF-α, IL-1β, and IL6 in rats with GHSR gene knock-out [40]. Thus, there is increasing evidence of the mediating role of the ghrelin/GHSR system in regulating pro-inflammatory cytokine release events in depressive disorders.

On the other hand, we can note that inflammation reduces neuroplasticity by downregulating brain-derived neurotrophic factor (BDNF), which may be the basis of the pathophysiology of depression [41,42]. IL-6 has been shown to be a reliable positive predictor of BDNF in patients with melancholic MDD [43]. However, the correlation between inflammation and BDNF requires further study. Moreover, the ghrelin/GHSR system can also produce antidepressant effects through the regulation of BDNF, which we will elaborate on below.

### 2.3. The Ghrelin/GHSR System Promotes Neurogenesis in Depression

Numerous clinical studies have shown that depression is closely associated with decreased size and function of the hippocampus and amygdala in limbic brain regions, and that patients with depression have reduced hippocampal volume and decreased neurogenesis [44,45], where neurogenesis is closely related to the treatment of depression. Studies have found that ablation of hippocampal nerve genesis in mice impairs the efficacy of antidepressants [18]. In animal experiments, antidepressant drugs promote hippocampal neurogenesis. Similarly, alterations in adult hippocampal neurogenesis mediate the effects of antidepressants, and chronic administration of the latter enhances the former [46].

As a neuropeptide, ghrelin exhibits strong neuroprotective effects. In rat cortical neuronal damage induced by hypoxia and hypoglycemia [47], ghrelin inhibited the neuronal damage process, and the protective effect disappeared after administration of a GHSR-specific inhibitor. In addition to this, a reduction in oligodendrocyte and neuronal apoptosis was found in an experiment in which ghrelin was administered after spinal cord injury in rats [48]. Most notably, the antidepressant effects exerted by ghrelin in vivo can be dependent on its neuroprotective effects. The published literature shows that ghrelin directly increases hippocampal neurogenesis in the treatment of depression [49]. Further studies have identified hippocampal neuroprotection as the primary mechanism by which a stress-induced increase in ghrelin protects the organism from the stress-induced worsening of associated depression [50]. In addition to the protective effects of ghrelin on hippocampal neurons against depression, the activation of catecholaminergic neurons has also been identified as a possible mechanism contributing to the antidepressant effects of ghrelin [51]. Although numerous studies have demonstrated that ghrelin exerts its antidepressant effects through neuroprotection, the mechanisms involved are currently not well understood. It has been found that in the hippocampus, ghrelin is able to cross the BBB and bind to GHSR1a to improve cognitive function and enhance hippocampal neurogenesis [52], and it can enhance LTP [53]. Ghrelin also directly induces the proliferation and differentiation of adult neural progenitor cells in the hippocampal subgranular zone. The number of progenitor cells was reduced in the GHSR1a KO mouse compared with the wild-type controls [49]. In addition to this, another report concluded that ghrelin provides neuroprotection through the activation of AMPK and enhances the clearance of damaged mitochondria [54], and it has also been demonstrated that ghrelin mediates neuroprotection through the inhibition of glial cell activation and the release of pro-inflammatory mediators [55]. In addition to the above-mentioned related mechanisms, it has been experimentally confirmed that BDNF plays an important role in the pathophysiology of depression [56]. The antidepressant effect exerted by ghrelin by regulating the relevant expression of BDNF is also a hot topic of research.

### 2.4. The Regulation of Astrocyte Physiology by the Ghrelin/GHSR System

Analysis of post-mortem tissue from patients with affective disorders has revealed a decreased number of astrocytes in several brain areas [57]. In addition, chronic unpredictable mild stress (CUMS)-induced cellular, metabolic and behavioral deficits can be reversed with antidepressants, which promotes glial cell Glu clearance [58]. Another investigation reported that the long-term administration of the antidepressant fluoxetine reversed the stress-induced decline in the number of hippocampal glial cells in tree shrews, and the relevance of the structural plasticity of astrocytes in stress and therapeutic support with antidepressants was proposed [59]. These results all suggest that antidepressant-mediated changes in astrocytes may be key to their effects and that these cells may play an active role in brain function that is connected to the process of depression.

Traditionally, astrocytes have often been thought of as brain glue, a class of cells that only provide metabolic and functional support to neurons. However, with the discovery of various neurotransmitter receptors and channels on the astrocyte membrane, our understanding of the function of astrocytes in the nervous system has fundamentally changed [60]. A large number of studies have found that the most prominent neurotransmitter receptors expressed on astrocyte membranes are GPCRs, including metabotropic glutamate receptors, adrenergic receptors, GABAergic receptors, cholinergic receptors, histaminic receptors, dopaminergic receptors, and neurotrophic factor receptors [61,62,63]. Upon activation of these receptors by neurotransmitters released by presynaptic neurons, astrocytes can undergo “gliotransduction” [64], releasing gliotransmitters to feed back on neuronal excitability and synaptic transmission [65,66]. Thus, astrocytes are now considered to be active participants in neuronal communication.

GHSR1a has been shown to be expressed in astrocytes in the arcuate nucleus of the hypothalamus [67] and the dentate gyrus of the hippocampus [68]. Therefore, exploring whether ghrelin is associated with astrocytes will be very interesting. A study found that the astrocytoma cell line C6 could respond to GHRP-6 by upregulating GHSR1a levels, increasing the activation of the PI3K/Akt pathway and its own proliferation [69]. Moreover, this effect can be inhibited by D-Lys3-GHRP-6, an antagonist of GHSR1a [68]; so, ghrelin may exert neuroprotective effects by stimulating astrocyte proliferation through GHSR1a to increase the expression of the PI3K/Akt pathway. However, another study found that ghrelin did not show a promotive effect on astrocyte proliferation and that it reversed the activation and accumulation of astrocytes in hippocampal neurodegeneration following hippocampal excitotoxicity injury induced by sea manate [55]. This difference may be related to the activation of astrocytes and the increase in the concentrated release of pro-inflammatory cytokines in inflammatory and related conditions and the triggering of neuroinflammation [70]. In conclusion, the specific mechanisms associated with ghrelin and astrocytes need to be investigated more thoroughly, as ghrelin may have a neuroprotective role in the development of depression through the regulation of astrocyte physiological activity by GHSR1a.

### 2.5. The Role of the Ghrelin/GHSR System in Endocrine Disruption in Depression

Depression has long been recognized as having a correlation with endocrine disruption, with the overactivity of the hypothalamic–pituitary–adrenal (HPA) axis being the most common trait [71]. In addition, the hypothalamic–pituitary–thyroid (HPT) axis and the hypothalamic–pituitary–gonadal (HPG) axis are also disturbed in depressed patients [72,73]. Therefore, changes in the levels of related hormones in the body can provide ideas for research on the treatment of depression. For example, testosterone levels are reduced in men suffering from depression [74], and the vulnerability of perimenopausal women to depression is associated with changes in estrogen [75]. Then, we can consider testosterone and estrogen to be targets for further research on antidepressants.

There is growing evidence that ghrelin is involved in the regulation of endocrine disruption in depression. For example, ghrelin can activate hypophysiotropic corticotropin-releasing factor (CRF) neurons and the HPA axis via inhibition of local GABAergic tone, in an arcuate nucleus (ARC)-independent manner [76,77]. Sarah et al. found that ghrelin suppressed anxiety after acute stress by stimulating the HPA axis at the level of the anterior pituitary [78]. However, des-acyl ghrelin administration has differential effects on the HPA axis and anxiety-like behavior in ghrelin-oacyltransferase (GOAT) KO mice and ghrelin KO mice [79]. Des-acyl ghrelin produces anxiolytic effect under stress, but GOAT KO mice showed anxiety-like behavior under stressed conditions. Ghrelin also affects the activity of the HPT axis by decreasing thyroid-stimulating hormone and increasing free thyroxine in the plasma [80]. In addition, the β1-adrenergic receptor blocker atenolol exacerbated depression-like behaviors in chronic social defeat stress (CSDS) mice by attenuating the increase in plasma acylgrowth factor-releasing peptide [81]. In conclusion, the relevant role of ghrelin in endocrine disruption in depressed patients requires more research.

## 3. Signaling Pathways Induced by Ghrelin/GHSR1a System in Depression

### 3.1. cAMP/CREB/BDNF Signaling Pathway

Neurodegenerative and neuropsychiatric disorders can be caused by inadequate supply of neurotrophic factors [82]. Among them, BDNF, as a member of the neurotrophic protein family, plays extremely important roles in promoting neuronal growth, survival, and differentiation; in synaptic transmission; in enhancing central plasticity [83,84,85]; and in slowing down depressive progression. Relatedly, ghrelin increases total BDNF mRNA expression in the mouse hippocampus and synthesizes different kinds of BDNF mRNAs by acting on different promoters in rats of different ages [86]. Different BDNF transcripts exhibit different subcellular localization that selectively shape the proximal and distal compartments of the cytosol or dendrites [87] and play an important role in increasing neuronal plasticity [88].

**Figure 1:**
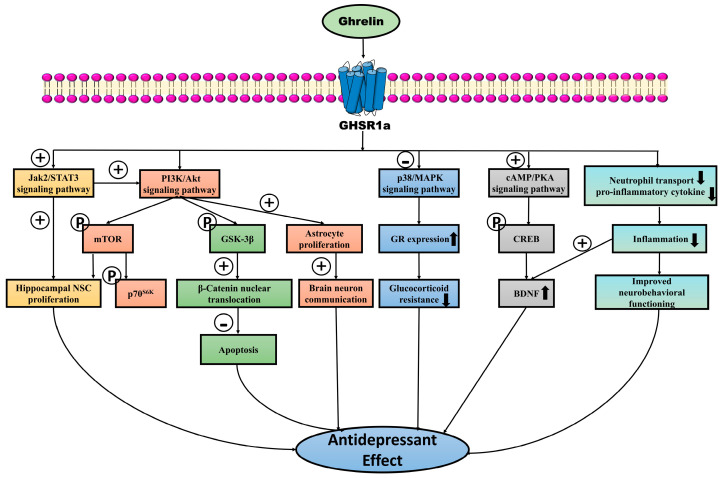
The antidepressant potential of ghrelin, which exerts neuroprotective effects by promoting neuronal proliferation and neurotrophic factor production in the brain. Ghrelin can inhibit apoptosis, reduce inflammation, increase astrocyte production, and significantly inhibit the process of depression. Ghrelin interacts with GHSR1a to increase the proliferation of hippocampal NSCs by increasing the expression of the Jak2/STAT3 signaling pathway, which also indirectly upregulates the PI3K/Akt/mTOR pathway to jointly exert neuronal protective effects. In addition, ghrelin binding to its receptor can also increase the expression of the PI3K/Akt signaling pathway, play a pro-neural cell proliferation role by promoting the phosphorylation of downstream molecules (mTOR and p70S6K), and inactivate GSK-3β by promoting GSK-3β phosphorylation, which can further increase the nuclear translocation of β-catenin, thus decreasing cellular apoptosis, playing a role in promoting cell survival. The activation of this pathway also promotes the proliferation of astrocytes and the communication of neurons in the brain. Moreover, ghrelin treatment can inhibit the p38-MAPK signaling pathway, increase GR expression, and inhibit glucocorticoid resistance in depressed patients. Additionally, ghrelin increases CREB phosphorylation by promoting the expression of the cAMP/CREB signaling pathway, thus regulating BDNF transcription and eventually exerting neuroprotective effects to inhibit depression. Besides this, ghrelin levels increase in the regulation of inflammatory response; further, this hormone downregulates neutrophil transport and the amount of pro-inflammatory cytokines, thus reducing inflammation, and it also increases the expression of BDNF and improves neurobehavioral function. In the figure, the subtraction symbol indicates inhibitory/suppressing action, while the addition symbol indicates stimulatory action. The P labeled in the circle represents substrate phosphorylation. ↑, increase; ↓, decrease.

Impaired cAMP signaling occurs in patients with major depression. In addition, in the hippocampus and prefrontal cortex of patients, the levels of BDNF, CREB, and p-CREB are significantly reduced [89,90,91,92], and so are the levels of BDNF mRNA in peripheral monocytes in this population [93]. Interestingly, the central administration of ghrelin normalized hippocampal BDNF levels [94]. In addition, exogenous ghrelin can improve depressive behavior by upregulating CREB signaling through the activation of ghrelin receptors and the cAMP/PKA signaling pathway and by increasing BDNF expression downstream [24]. Thus, ghrelin-induced increases in BDNF in the hippocampus involve the activation of the GHSR1a/cAMP/PKA/CREB signaling pathway (Figure 1).

### 3.2. p38-MAPK Signaling Pathway

Several studies have demonstrated that p38-MAPK is activated in response to various stressful stimuli and is involved in the pathologic process of depression [95,96]. p38-MAPK can be activated by interferon and lipopolysaccharide to upregulate the expression of the depression-related gene IDO [97,98], and it can also exacerbate esophageal cancer-associated depression by directly enhancing the expression of the IDO gene [99]. Therefore, the expression status of p38-MAPK-pathway-related substances could be a powerful tool for depression monitoring, while p38-MAPK itself could be a target for antidepressant research. In addition, the activation of the p38-MAPK pathway phosphorylates glucocorticoid receptor (GR), whose phosphorylation is associated with reduced glucocorticoid sensitivity [100], which may be closely related to the glucocorticoid resistance exhibited by depressed patients [101]. In contrast, ghrelin treatment in rats activates GHSR1a and decreases p38-MAPK phosphorylation, which, in turn, increases the GR levels [102]. Furthermore, no significant increase in the phosphorylation of p38 by CSDS in vector-treated mice was observed after ghrelin treatment. Additionally, hippocampal GHSR-deficient mice showed higher levels of p38 phosphorylation than control mice, suggesting that ghrelin may also mediate antidepressant mechanisms by inhibiting the p38-MAPK signaling pathway in the hippocampus [103]. Interestingly, social failure stress produced depression-like behavior in wild-type mice, but the selective deletion of p38-MAPK in the serotonergic neurons of the nucleus dorsalis of the mouse middle suture protected the mice under stress induction [104]. This suggests that p38-MAPK has the ability to specifically regulate selected downstream targets; thus, the role played by this pathway in antidepressant disorders requires further investigation.

### 3.3. PI3K/Akt Signaling Pathway

Depression is also closely related to neurogenic hypoplasia [105], and PI3K/Akt is thought to be an important signal for the proliferation of adult hippocampal progenitor cells [106]. Akt can exert its utility in controlling cellular proliferation by activating the phosphorylation of its downstream targets, GSK-3β, mTOR, and p70^S6K^, where GSK-3β is a pro-apoptotic protein whose activity plays an important role in neuropathology and psychiatric disorders [107]. β-Catenin, as a transcription factor regulated by GSK-3β, undergoes nuclear translocation under conditions of GSK-3β inactivation [108], which is an indispensable step in its role in promoting cell survival. In addition, downstream of PI3K/Akt, the phosphorylation of mTOR and p70^S6K^ also promotes the proliferation of neural stem cells [109]. Interestingly, ghrelin could induce hippocampal neural stem cell (NSC) proliferation by activating the PI3K/Akt signaling pathway by binding to GHSR1a, and the stimulatory effects of ghrelin on GSK-3β, mTOR, and p70^S6K^ phosphorylation were significantly inhibited by treatment with GHSR1a-specific antagonist D-Lys-3-GHRP-6 [110]. Furthermore, it has been demonstrated that ghrelin enhances the nuclear translocation of β-catenin, which, in turn, contributes to its anti-apoptotic effects [111]. Therefore, we may hypothesize that ghrelin may promote neuronal cell proliferation through the activation of the PI3K/Akt pathway and subsequently play a role in the treatment of depression. Moreover, autophagy plays an important role in maintaining neuronal stem cells and adult neuronal plasticity, while ghrelin can stimulate autophagy by inhibiting the PI3K/AKT/MTOR signaling pathway [112]. However, an experiment in a mouse model of corticosterone-induced depression found that overactive neuronal autophagy depleted BDNF and impaired adult hippocampal neurogenesis [84]. Therefore, it remains to be investigated whether ghrelin can regulate autophagy homeostasis in vivo through the PI3K/AKT/MTOR pathway and promote neurogenesis.

### 3.4. Jak2/STAT3 Signaling Pathway

The Jak2/STAT3 signaling pathway, like the aforementioned PI3K/Akt signaling pathway, has also been shown to play an important role in neuroprotection. Unlike single pathways that act independently, one study found that resveratrol may exert neuronal protection by indirectly upregulating the PI3K/Akt/mTOR pathway through the activation of Jak2/STAT3 [113]. As shown in Figure 1, the exposure of rat hippocampal NSCs to the Jak2/STAT3 inhibitor cucurbitacin I significantly blocked the proliferative effects of ghrelin on NSCs [110]. Thus, ghrelin could also be potent through the activation of the Jak2/STAT3 pathway. Furthermore, this pathway not only plays an active role in neuroprotection and regeneration, but its role in neuroinflammation has also been shown to be promoted. It has been found that the inhibition of the Jak2/STAT3 pathway ameliorates neuroinflammation [114] and reduces neuronal senescence by suppressing the inflammatory response [115]. Since depression is closely related to decreased neuronal genesis and the upregulation of inflammatory factors and given that the activation of the Jak2/STAT3 pathway has been found to have completely opposite effects in these two aspects, whether this pathway can be a therapeutic target for depression needs to be investigated at a deeper level in terms of its multifaceted mechanisms. The study of whether ghrelin regulates neuroinflammation when activating the Jak2/STAT3 pathway will be an important basis for comprehensively determining whether ghrelin can be used as a target for the treatment of depression.

## 4. Ghrelin/GHSR as a Therapeutic Target for Depressive Disorder

Many neuropeptides have been reported as targets for depression treatment. For example, nonselective glycopeptide receptor agonists (galnon) can exert antidepressant effects in preclinical models of depression [116,117]. VP antagonists have shown similar antidepressant behavior in preclinical studies [118,119]. Similarly, many studies have shown that ghrelin can be used as a powerful tool in the treatment of depression. It has been reported that ghrelin produces antidepressant effects in estrogen-deficient mice [94] and that it may also counteract depressive symptoms caused by chronic stress [120]. In addition, many substances that exert antidepressant effects by modulating ghrelin/GHSR expression have been identified. For example, paeoniflorin (PF) significantly increased the expression of GHSR1a to mediate antidepressant effects [121], and the GHSR inhibitor JMV29282259 blocked the saffronin-induced expression of neuroplasticity-related proteins [122]. Here, we briefly summarized the correlation between the ghrelin/GHSR system and depression, as well as the research methodology, as shown in Table 1. Thus, the ghrelin/GHSR system has many potent functions in the defense against depression-like symptoms. However, further studies are needed because of the stable antidepressant behavioral effects of neuropeptides expressed in various tests and the two-sided nature of ghrelin’s effects on depression in different models [123]. Recently, liver-expressed antimicrobial peptide 2 (LEAP2) was reported as an endogenous antagonist of GHSR and produced in the liver and small intestine [124,125]. Whether LEAP2 is involved in the development of depression needs further study.

## 5. Conclusions and Future Direction

There is an ongoing debate about the role of ghrelin in depression. The majority of studies suggest that ghrelin has antidepressant effects, with few studies indicating depressogenic effects. Indeed, ghrelin/GHSR can exert antidepressant and neuroprotective effects by triggering multiple signaling pathways, including cAMP/CREB/BDNF, PI3K/Akt, Jak2/STAT3, and p38-MAPK. Moreover, GHSR1a can also form dimers with other GPCRs to exert antidepressant effects. Therefore, the ghrelin/GHSR system is becoming a new target for the treatment of depression. However, there are two-sided claims on ghrelin’s antidepressant potency according to different experiments; for instance, ghrelin had no antidepressant effect on young rats, and the drug’s neuropharmacology differed in adolescents and adults. Future experiments should, therefore, focus on investigating the link between the pathogenesis of ghrelin and depression in different depressive groups, where the development of receptor-biased drugs is also a good strategy. Moreover, more studies are necessary to determine the extent to which central and peripheral ghrelin signaling are functionally interconnected, as this understanding is crucial to the development of new ghrelin-based therapeutic agents. Finally, promising findings from animal studies necessitate further human-based research to ascertain the extent to which such results can be applied to human disorders.

## Figures and Tables

**Table 1:** Effects of ghrelin/GHSR system on MDD in rodents.

Animal and Stress Paradigm	Behavioral Test	Intervention	Signal Molecules	Effects	References
Mouse CSDS	FST and TST	GHSR1a knock-out	BDNF ↓, IL-6 ↑	Pro-depression effect	[26]
Rat	FST and TST	i.c.v. injection of ghrelin	HPA ↑	Immobility time ↑ in TST	[126,127]
Male C57BL/J6 mice, RS	TST, OFT, and FST	rAAV-Mediated overexpression of GHSR1a	c-Fos ↑	Antidepressant-like effect	[128]
Male mice, restraint stress	EPM	Ghrelin KO	pERK ↓	Decreases anxiety-like behavior	[129]
C57BL/J6 mice, prenatal stress	OFT, TST, FST, and SPT	i.p. injection of crocin	PI3K/Akt ↑ MTOR ↑	Antidepressant-like effect	[122]
Male SD rats, HFD and DDR	FST, OFT, and EPM	Intra-VTA administration of ghrelin	TNF-α ↓ IL-1β↓ IL-6 ↓	Alleviates depression-like behavior	[130]
Male SD rats, CAO	SPT, OFT, and EPM	Subcutaneous injection of ghrelin	Iba-1 ↓ GFAP ↓	Attenuates depression-like behavior	[131]
Male C57BL/J6 mice, CSDS	SIT, FST, OFT, and EPM	Intrahippocampal ghrelin infusions, AAV-siRNA of GHSR1a	p38-MAPK ↓	Antidepressant effect	[103]
Male C57BL/J6 mice	TST and FST	Lateral ventricle injection of ghrelin	CREB ↑ BDNF ↑	Improves cognition and antidepressant-like effects	[24,94]
Adult female mice, OB	OFT and TST	Ghrelin into the hippocampus	MAPK ↑ CaMKIIa ↑	Antidepressant-like effects	[25]
Male C57BL/J6 mice, CUMS	OFT, EPM, and FST	Intraperitoneal (i.p.) injection of ghrelin, GHSR knock-down	PI3K/Akt ↑ CDK2 ↑ CyclinD1 ↑	Spine density ↑, proliferation of hippocampal NSCs, and neurogenesis ↑	[132]

The upward arrow indicates increase; The downward arrow indicates decrease.

## Abbreviations

BBB, blood–brain barrier; BDNF, brain-derived neurotrophic factor; CAO, coronary artery occlusion; CDK, cyclin-dependent kinases; CNS, central nervous system; CREB, cAMP response element-binding protein; CSDS, chronic social defeat stress; CUMS, chronic unpredictable mild stress; DDR, disturbed diurnal rhythm; EPM, elevated plus maze; FST, forced swimming test; GFAP, glial fibrillary acidic protein; GHSR, growth-hormone-releasing peptide receptor; GSK-3β, glycogen synthase kinase 3β; GR, glucocorticoid receptor; HFD, high-fat diet; HPA, hypothalamic–pituitary–adrenal; HPT, hypothalamic–pituitary–thyroid; HPG, hypothalamic–pituitary–gonadal; Iba-1, ionized calcium-binding adapter molecule 1; LH, luteinizing hormone; MDD, major depressive disorder; mPFC, medial prefrontal cortex; mTOR, mammalian target of rapamycin; NSCs, neural stem cells; OFT, open field test; OX1R, orexin 1 receptor; OB, olfactory bulbectomy surgery; p70^S6K^, p70 ribosomal protein S6 kinase; RS, restraint stress; SIT, social interaction test; SPT, sucrose preference test; TST, tail suspension test.
